# *In vitro* biocompatibility study of sub-5 nm silica-coated magnetic iron oxide fluorescent nanoparticles for potential biomedical application

**DOI:** 10.1038/srep46513

**Published:** 2017-04-19

**Authors:** Sabrina Foglia, Mario Ledda, Daniela Fioretti, Giovanna Iucci, Massimiliano Papi, Giovanni Capellini, Maria Grazia Lolli, Settimio Grimaldi, Monica Rinaldi, Antonella Lisi

**Affiliations:** 1 Institute of Materials for Electronics and Magnetism (IMEM), Department of Engineering, ICT and technologies for energy and transportation, National Research Council (CNR), Parma, Italy; 2 Institute of Translational Pharmacology (IFT), Department of Biomedical Sciences, National Research Council (CNR), Rome, Italy; 3 Department of Science, University Roma Tre, Rome, Italy; 4 Institute of Physics, Catholic University of the Sacred Heart, Rome, Italy

## Abstract

Magnetic iron oxide nanoparticles (IONPs), for their intriguing properties, have attracted a great interest as they can be employed in many different biomedical applications. In this multidisciplinary study, we synthetized and characterized ultrafine 3 nm superparamagnetic water-dispersible nanoparticles. By a facile and inexpensive one-pot approach, nanoparticles were coated with a shell of silica and contemporarily functionalized with fluorescein isothiocyanate (FITC) dye. The obtained sub-5 nm silica-coated magnetic iron oxide fluorescent (sub-5 SIO-Fl) nanoparticles were assayed for cellular uptake, biocompatibility and cytotoxicity in a human colon cancer cellular model. By confocal microscopy analysis we demonstrated that nanoparticles as-synthesized are internalized and do not interfere with the CaCo-2 cell cytoskeletal organization nor with their cellular adhesion. We assessed that they do not exhibit cytotoxicity, providing evidence that they do not affect shape, proliferation, cellular viability, cell cycle distribution and progression. We further demonstrated at molecular level that these nanoparticles do not interfere with the expression of key differentiation markers and do not affect pro-inflammatory cytokines response in Caco-2 cells. Overall, these results showed the *in vitro* biocompatibility of the sub-5 SIO-Fl nanoparticles promising their safe employ for diagnostic and therapeutic biomedical applications.

In the last years, an increasing attention and interest on nanobiotechnological materials, nanoparticles (NPs) in particular, have emerged as a promising tool in the field of nanomedicine applications^1^,^2^,^3^,^4^,^5^,^6^.

NPs are used for diagnosis, prevention and treatment of diseases as much as for tissue engineering and regenerative medicine applications. These implementations demand the cross communication among different disciplines for the success of new therapies in restoring and regenerating the normal function of damaged cells, organs and tissues^7^,^8^,^9^,^10^.

Nanoparticles made up of components between 1 nm and 100 nm in size and specifically magnetic iron oxide nanoparticles (IONPs), approved by Food and Drug Administration (FDA)^11^, have been extensively studied and have attracted much interest for their intriguing properties employable in a wide range of biomedical applications (Fig. 1).

The synthesis strategy used to obtain IONPs represents an important challenge to consider carefully. For this scope, the commonly used method is the reverse micelle synthesis in nonpolar organic solvents that allows the synthesis and production of small-sized and uniform nanoparticles. As they are only organic-soluble^12^,^13^, their use is limited and not compatible for biomedical applications. The best approach to overcome this difficulty is the post-synthetic modification method by co-precipitation in an alkaline aqueous solution^14^.

To cover the IONPs cores with an inert material, such as silica, is essential to obtain a passive biocompatible coating, in order to improve the biological application and chemical stability of the nanoparticles. It prevents their aggregation and degradation and grants their monodispersion^14^ also increasing surface functionalization which allows the reaction and binding of specific ligands such as drug molecules, fluorescent compounds and biological agents^4^,^9^. Biocompatibility assessment of silica coated nanoparticles performed in human vascular endothelial cells (HUVEC) and cancer cells derived from the cervix carcinoma (HeLa) showed cell type dependent nanotoxicity^15^. On the other hand, other studies reported no toxicity or dose-dependent effects in primary human cells^16^ as well as size-dependent effects^17^.

Size is another factor which plays an important role in the IONPs applications. Regarding this, several studies have been done to control and reduce it^18^,^19^. Size strongly affects the number of active sites, the superparamagnetic characteristics^19^, the retention and biodegradation time, and the tissue biodistribution^18^,^20^.

As a matter of fact, when surface/volume ratio increases, the number of active sites is enhanced suggesting that, by using smaller IONPs (less than 5 nm diameter) to carry ligands, the process could become more efficient^18^,^19^.

Furthermore, in therapeutic applications, smaller IONPs offer prolonged blood circulation, they are optimal for cross capillary walls^14^ and have the most effective tissue distribution, especially in tumour regions^21^,^22^,^23^,^24^,^25^,^26^. The superparamagnetic behaviour^9^,^27^ of small-sized nanoparticles can be used for attracting them by an external magnetic field to the tissue of interest thus allowing efficient magnetically mediated gene/drug targeting and/or hyperthermia^28^,^29^,^30^,^31^.

The size is one of the main factors that can determine the IONP cellular internalization process^18^,^32^. Size, surface charge, and functional groups on the shell can affect cell^18^,^32^,^33^,^34^,^35^,^36^ internalization and subcellular distribution, processes still under debate.

In this work, we report ultrafine superparamagnetic nanoparticles (average core size of 3 nm), water-dispersible, prepared by an “arrested precipitation strategy”. By a one-pot approach, nanoparticles were coated with silica to prevent their degradation/aggregation and to increase their surface functionalization, and contemporarily labelled with fluorescein isothiocyanate (FITC) dye to visualize their intracellular localization.

The resulting new sub-5 nm silica-coated magnetic iron oxide fluorescent (sub-5 SIO-Fl) nanoparticles were tested in CaCo-2 cell line, a well characterized model of the intestinal epithelium^37^, commonly used for biopharmaceutical evaluations^38^ as well in toxicity studies either as differentiated or undifferentiated cells^39^,^40^,^41^,^42^. The gastrointestinal tract, as the lung and skin, is considered the main way through nanoparticles may access the body^43^,^44^,^45^ and the interaction of nanoparticles with the intestinal epithelium regulate their systemic absorption and organ-specific toxicity. Furthermore, in this paper we tested the sub-5 SIO-Fl on undifferentiated CaCo-2 cells, due to their higher sensitivity to nanoparticles compared to differentiated ones^40^.

We studied sub-5 SIO-Fl nanoparticles cellular uptake and intracellular localization. Furthermore, we investigated if their uptake affected CaCo-2 cell morphology, growth, viability, cell cycle distribution, as well as transcriptional, translational and secretory activities, in a dose-dependent manner. To further shed light on their biocompatibility, the effect of the sub-5 SIO-Fl nanoparticles on CaCo-2 cell differentiation and pro-inflammatory response was analysed.

Our results demonstrated that the nanoparticles, synthesized as described in the present work, are *in vitro* biocompatible and non-toxic, promising safe application in diagnostics and therapeutics.

## Results and Discussion

### Synthesis and characterization of 3 nm superparamagnetic nanoparticles

Magnetite nanoparticles were synthesized by an “arrested precipitation strategy” following reported procedure^46^. The particles were prepared by coprecipitation in aqueous solution of Fe^3+^ and Fe^2+^ ions (details are provided in Supplementary Methods) and characterized for size and shape, crystallographic structure and magnetic properties by transmission electron microscopy (TEM) analysis, Dynamic light scattering (DLS), X-ray diffraction analysis (XRD) and Magnetization measurements respectively (see Supplementary Methods).

TEM images of 3 nm superparamagnetic nanoparticles as-synthesized indicates spherical shape. Nanoparticles diameter has been quantified from TEM image analysis (3 ± 1 nm) and from DLS analysis (3.6 ± 0.4) (see Supplementary Fig. S1).

XRD analysis shows that the nanocrystals are highly crystalline Fe_3_O_4_ magnetite (see Supplementary Fig. S2). Furthermore, the magnetization measurements demonstrated the finite-size effect and the results showed that nanoparticles as-synthesized are superparamagnetic at room temperature (see Supplementary Fig. S3).

### Synthesis and characterization of sub-5 nm silica-coated magnetic iron oxide fluorescent nanoparticles

The nanoparticles of Fe^3+^ and Fe^2+^ ions were contemporarily coated with a thin silica shell and covalently attached with FITC dye since, in a biological context, its photophysical properties on one hand eludes dye toxicity avoiding dye leaching^47^ and on the other hand allows its visualization inside the cells. The preparation of sub-5 SIO-Fl nanoparticles was performed through a new one pot synthetic strategy modifying the procedures reported by Z. Lu *et al*., A.P. Philipse *et al*. and A. van Blaaderen *et al*.^48^,^49^,^50^. A colloidal dispersion of FICT was obtained covalently attaching the dye to a coupling agent, aminopropyltriethoxysilane (APS), due to the addition reaction of the amine with the thioisocyanate group. A magnetite ferrofluid was prepared suspending Fe_3_O_4_ nanopowder in an ethanol solution of tetraethyl orthosilicate (TEOS) with ammonia. The fluorescent suspension was mixed with the ferrofluid, allowing the growth of the dye shell on the magnetic surface, due to the silica linkage obtained in a single step.

**Figure 2:**
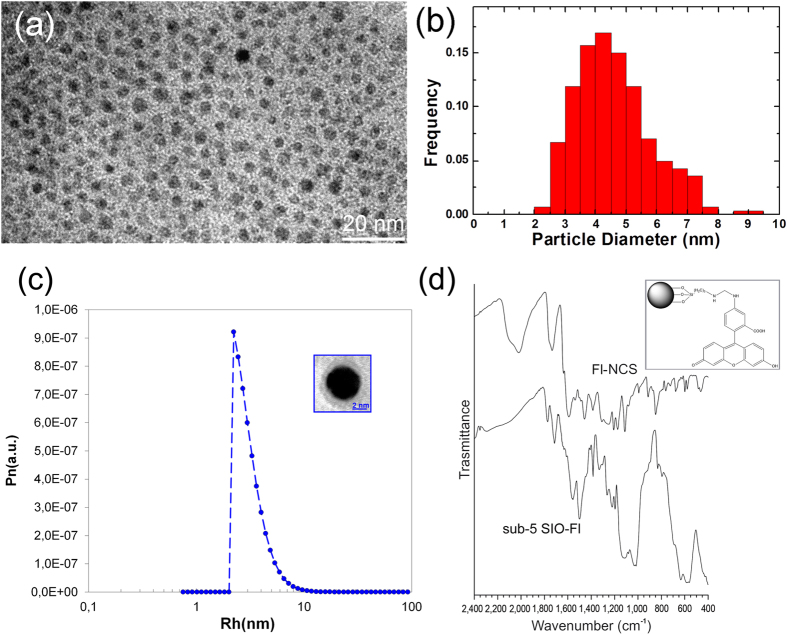
Characterizations of sub-5 nm silica-coated magnetic iron oxide fluorescent nanoparticles. TEM image (**a**) and the statistical distribution of nanoparticles diameters (**b**) (sample size = 300 nanoparticles); size distribution obtained from DLS (**c**). The inset in (**c**) shows a representative high-resolution image of a single nanoparticle; FTIR spectra (**d**) in the 2400–400 cm^−1^ region of sub-5 nm silica-coated magnetic iron oxide fluorescent nanoparticles (sub-5 SIO-Fl) and fluorescein isothiocyanate (Fl-NCS). The inset in (**d**) is the Sketch of the proposed chemical structure of sub-5 SIO-Fl nanoparticles).

Morphological characterization of the sub-5 SIO-Fl nanoparticles recorded by TEM imaging indicates a spherical shape with a mean diameter of 4.8 nm (Fig. 2a). The histogram of particle size based on statistical analysis over 300 particles is reported in Fig. 2b and reveals the rather high monodispersity of the nanoparticles size prepared by the current procedure.

The dynamic light scattering (DLS) measurements also confirmed the nanoparticle size determined by TEM analysis (Fig. 2c). The diffusion coefficients of sub-5 SIO-Fl nanoparticles as obtained by DLS were independent of the scattering angle, indicating an unclustered dispersion (Fig. 2c). The calculated hydrodynamic diameter (D_H_) of 4.6 ± 0.5 nm agrees with the particles size determined by TEM.

Overall, all data so far showed that the nanoparticles synthesized and functionalized as described are homogeneous, highly uniform and fully retain their ferro-magnetic properties.

To analyze Protein Corona formation SIO nanoparticles have been incubated in complete cell culture medium for 1, 12, 24, 36, 48 and 60 hours, respectively, and the resulting SIO nanoparticle-protein corona complexes were separated from unbound proteins and excess medium by centrifugation and extensive washing (see Materials and Methods). As reported in Supplementary Table S1, after 1 hour exposure to complete cell culture medium, adsorption of proteins leads to larger complexes (D_H_ = 8.5 ± 2.2 nm). After 12 hours of incubation, the size of complexes reached its plateau value (D_H_ ≈ 9.4 nm). Zeta Potential displays a similar trend: from −15.5 mV (1 hour), it reaches its plateau after 12 hours (plateau value ≈ −9.2 mV).

For structural proof, we carried out a Fourier Transform Infrared Spectroscopy (FTIR) study of the sub-5 SIO-Fl nanoparticles (Fig. 2d). The spectra were recorded in the 2400–400 cm^−1^ region and the IR spectrum of pristine fluorescein isothiocyanate (Fl-NCS) is shown for comparison. Peak position and assignment is shown in Table 1.

In the spectrum of sub-5 SIO-Fl the Fe-O stretching band at 570 cm^−1 ^51 is evident, as well as the bands at 1110 cm^−1^ (Si-O-Si stretching) and 1030 cm^−1^ (Si-O-C, Si-OH stretching).

Moreover, bands related to the Fluorescein molecule are detected. The strong band at 2020 cm^−1^ with a shoulder at 2100 cm^−1^ in the Fl-NCS spectrum is related to the stretching of the NCS thiocyanate group; the band disappears in the sub-5 SIO-Fl spectrum confirming that fluorescein immobilization takes place via reaction between the isothiocyanate group and the terminal amino group of APS^52^.

Fluorescein can exist in solution either in dianionic (Fl^2−^) or monoanionic (Fl^−^) form depending on the pH solution; peak position and assignment in the range 1100–1600 cm^−1^ from literature data^53^ for Fl^−^ and Fl^2−^ is shown in Table 1. In the spectrum of pristine fluorescein, C=O stretching bands located at 1740 cm^−1^ (COOH groups) and at 1590 cm^−1^ (COO- groups) show that the carboxyl groups are partially protonated and partially deprotonated; partial carboxyl protonation is also confirmed by the C-OH stretching band located at 1264 cm^−1^. The overall spectrum of fluorescein consists of many vibrations related to the xanthene ring, whose assignment is shown in Table 1^53^,^54^. The FTIR spectrum of sub-5 SIO-Fl shows intense bands at 1740 cm^−1^ (protonated carboxyl C=O stretching) and 1264 cm^−1^ (C-OH stretching) while the carboxylate vibration located at 1590 cm^−1^ in the spectrum of fluorescein can no longer be detected. These data indicate that in sub-5 SIO-Fl the carboxyl group of fluorescein is predominantly in protonated form. The fluorescein bands in the wavenumber range 1180–1000 cm^−1^ cannot be detected being covered by the intense Si-O-Si and Si-O-C bands of the SiO_2_/APS layer; the other skeletal vibrations are found approximately in the same position as for pristine fluorescein. Infrared results are consistent with the structure of sub-5 SIO-Fl showed in the Fig. 2d inset. Sub-5 SIO-Fl nanoparticles result with monoanionic surface charge. Then, considering FITC as a hydrophobic dye^55^, FITC functionalization of sub-5 SIO-Fl nanoparticles results in the formation of hydrophobic groups on their surfaces. Hydrophobicity is required to enhance the interaction of nanoparticles with the cellular membrane and their uptake into cells. Cationic ligands, due to the high affinity with the cellular membrane that allows a high level of endocytic cellular uptake, can disrupt the cellular membrane, resulting in cytotoxicity by changing the cell membrane potential and intracellular concentration of calcium ions^55^,^56^. Therefore, non-cationic ligands enabling higher levels of cellular uptake of nanoparticles are in high demand for safety reasons^57^,^58^,^59^,^60^.

### Sub-5 nm silica-coated magnetic iron oxide fluorescent nanoparticles cell internalization and biocompatibility

Several publications demonstrate that iron oxide nanoparticles can exhibit significant cytotoxic effects on several cell lines while other studies reported little or no toxicity^61^,^62^,^63^.

The *in vitro* toxicity study is influenced by testing techniques, by treatment modalities and by the specific cell types used. In order to examine the feasibility of sub-5 SIO-Fl nanoparticles for nanomedicine applications we analysed their cellular internalization and biocompatibility in CaCo-2 cells at different concentrations.

### Cellular Uptake Study

**Figure 3:**
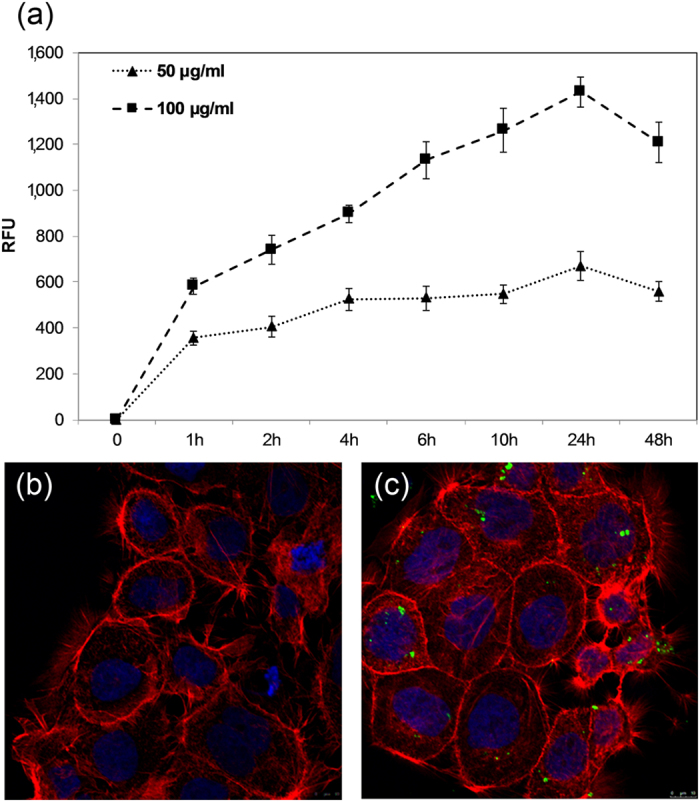
Cellular uptake and confocal image of CaCo-2 cells after incubation with sub-5 SIO-Fl nanoparticles. (**a**) Cellular uptake of sub-5 SIO-Fl nanoparticles by CaCo-2 cells. The cells were incubated with (▲) 50 μg/ml and (■)100 μg/ml of nanoparticles and the relative fluorescence emitted from the nanoparticles up taken by CaCo-2 cells was measured. Relative Fluorescence Unit (RFU) at both concentrations was reported as function of the incubation time. All data were expressed as the mean ± standard deviation (SD) (n = 3). (**b**,**c**) Confocal laser scanning microscopy study of sub-5 SIO-Fl nanoparticles cellular internalization (green) and rhodamine phalloidin-labelled F-actin analysis (red). (**b**) untreated cells; (**c**) cells after 48 h of exposure with 50 μg/ml of sub-5 SIO-Fl nanoparticles. A representative image of the cell middle confocal Z section shows the presence of nanoparticles inside the cells and their intracellular accumulation in the cytoplasmic region, around the nucleus. No difference in F-actin organization was reported between treated and untreated cells. Nuclei are counterstained with Hoechst (blue). Photographs were taken at a magnification of 40X.

Three different concentrations of sub-5 SIO-Fl nanoparticles (10 μg/ml, 50 μg/ml, and 100 μg/ml) were added to the culture medium of exponentially growing CaCo-2 cells and their cellular uptake was investigated measuring the relative fluorescence emitted (Fig. 3a). The uptake increased quickly in the first hours, reached a peak within 24 h and sub-5 SIO-Fl nanoparticles at 100 μg/ml exhibits higher uptake rate compared to sub-5 SIO-Fl nanoparticles at 50 μg/ml. When the experiments were performed by using 10 μg/ml of sub-5 SIO-Fl nanoparticles the relative fluorescence value was below the detection threshold.

These findings show that sub 5-SIO-Fl nanoparticles are quickly up taken by CaCo-2 cells, the process occurs in few hours and rich a pick within 24 h.

By TEM analysis, we also observed that the SIO nanoparticles were mainly localized freely within the cytosol of Caco-2 cells (Supplementary Fig. S5). Thus, we argue that they could diffuse across the plasma membrane, although we cannot exclude active endocytic mechanisms.

### Confocal Laser Scanning Microscopy Study

Sub-5 SIO-Fl nanoparticles cellular internalization was investigated by confocal laser scanning microscopy analysis on CaCo-2 cells treated with 50 μg/ml of nanoparticles after 48 h of exposure. The intracellular accumulation of sub-5 SIO-Fl nanoparticles distributed in the cytoplasmic region, around the nucleus, is shown in Fig. 3c. The F-actin organization of the treated cells was also analysed. F-actin is the most important and prevalent cytoskeletal protein for the cell shape, motility, endocytosis, traction force, division^64^, and its alteration can affect cellular functionalities. It has been reported that intracellular magnetic nanoparticles accumulation affects cell morphology and induce changes in the architecture of the actin cytoskeleton leading to a reduced capacity of cell proliferation and spreading^11^. No different F-actin organization was reported between the sub-5 SIO-Fl nanoparticles treated CaCo-2 cells and control ones, showing well-structured actin filaments that resulted concentrated around the cell membrane and in the external brush-like structures. Both control and treated CaCo-2 cells showed a polygonal morphology and a good substrate adhesion capability (Fig. 3b,c).

These results demonstrate that the nanoparticles as-synthesized are internalized and do not interfere with the CaCo-2 cells cytoskeletal organization nor with their cellular adhesion.

### Phase Contrast and Fluorescence Microscopy Study

Three different concentrations of sub-5 SIO-Fl nanoparticles (10 μg/ml, 50 μg/ml, and 100 μg/ml) were added to the culture medium of exponentially growing CaCo-2 cell and the cells were examined after 48 h of exposure by phase contrast and fluorescence microscopy analysis (details are provided in the Supplementary Methods).

**Figure 4:**
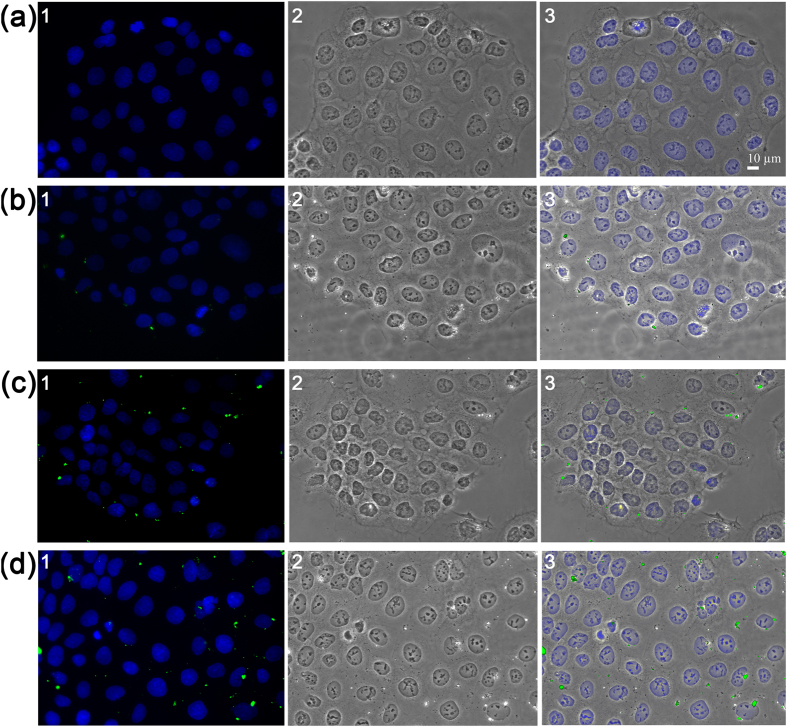
Phase Contrast and Fluorescence Microscopy Study of untreated and treated CaCo-2 cells. Fluorescence microscopy (first column), phase contrast (second column) and merged images analysis (third column) of CaCo-2 cells untreated (**a**) and treated for 48 h with sub-5 SIO-Fl nanoparticles (green) at 10 μg/ml (**b**), 50 μg/ml (**c**), and 100 μg/ml (**d**). Nanoparticles inside the cells unaffected CaCo-2 cell morphology, shape and adhesion. Nuclei are counterstained with Hoechst (blue). Photographs were taken at a magnification of 40X.

Treated cells resulted morphologically unaltered and well attached on the substrate, and the nanoparticles resulted distributed in the CaCo-2 cytoplasm compartment in a higher number accordingly with their higher concentration (Fig. 4b–d). These results provided evidence that the CaCo-2 cells treated with sub-5 SIO-Fl nanoparticles result unaffected in terms of cell morphology without evident shape deformations and nuclear structure changes, even when treated with the highest nanoparticles concentration (Fig. 4d). To examine the influence of sub-5 SIO-Fl nanoparticles on the CaCo-2 cell behaviour following long-term exposure, cell actin distribution was analysed after 7 days of treatment at the three nanoparticles concentrations above reported (see Supplementary Fig. S4). The fluorescence staining of rhodamine phalloidin-labelled F-actin (see Supplementary Methods) in treated CaCo-2 cells showed the same actin filament distribution with a typical sub-apical cytoskeletal localization, concentrated around the cell membrane, compared to control ones.

### Cytotoxicity Study

**Figure 5:**
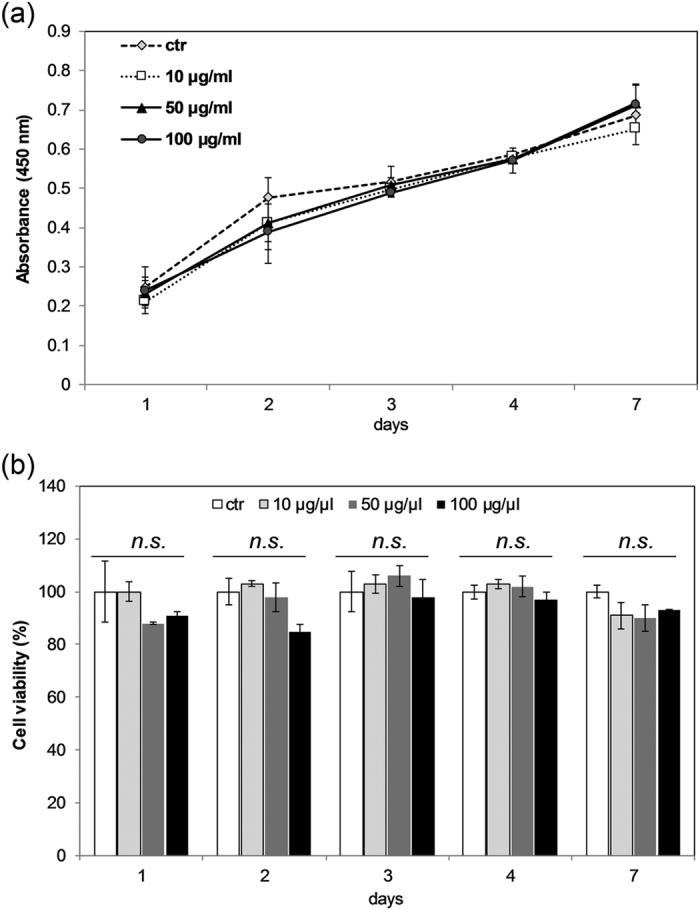
Sub-5 SIO-Fl nanoparticles cytotoxicity study. Cell proliferation (**a**) and cell viability (**b**) were analysed by BrdU incorporation assay and WST metabolic activity respectively in CaCo-2 cells treated with 10 μg/ml, 50 μg/ml, and 100 μg/ml of nanoparticles at 24, 48, 72, 96 hours, and 7 days after exposure. Cell viability was calculated as a percentage with respect to the control cultures (set to 100%). All data were expressed as the mean ± standard deviation (SD) (n = 3) (n.s., not significant).

Considering that the nanoparticles cytotoxicity is a crucial issue for nanomedicine applications we further investigated whether sub-5 SIO-Fl nanoparticles exhibited cellular toxicity when internalized in CaCo-2 cells model. Cell proliferation and cellular viability were studied in CaCo-2 cells treated with three different nanoparticles concentrations (10 μg/ml, 50 μg/ml and 100 μg/ml), and grown up to 7 days (Fig. 5). Treated cells showed the same increasing exponential trend compared to control ones with no significant changes in the proliferation rate (Fig. 5a). CaCo-2 treated cells also showed the same cellular viability as in the untreated control cells up to 7 days at all nanoparticles concentrations used (Fig. 5b).

### Cell cycle distribution

**Figure 6:**
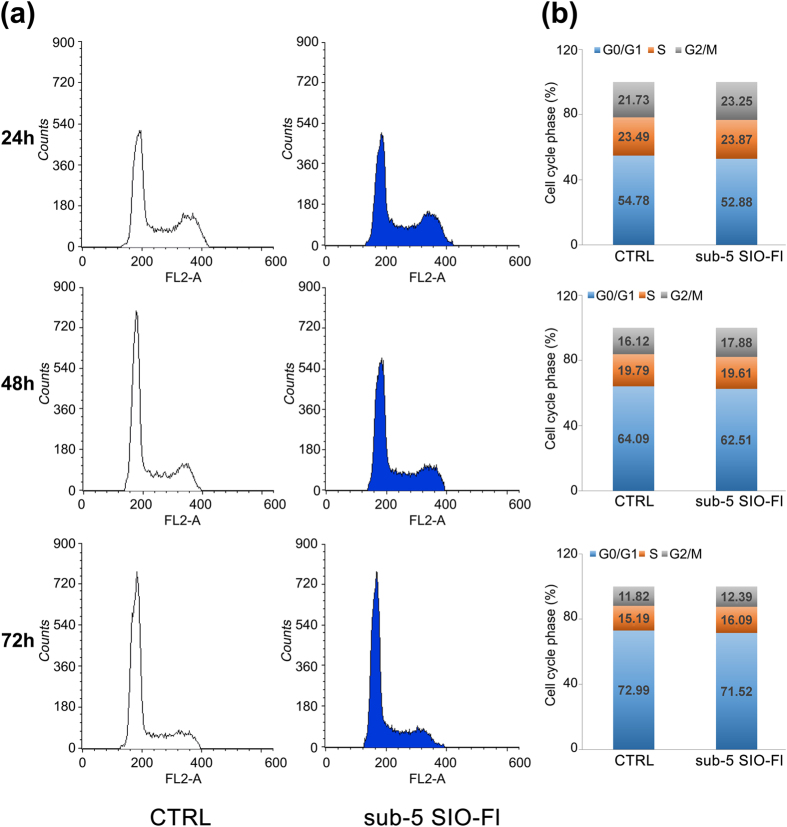
Cell cycle analysis in CaCo-2 cells. (**a**) Representative histograms of treated cells (sub-5 SIO-Fl group) with no alterations in cell cycle progression upon exposure to 100 μg/ml of nanoparticles for 24, 48 and 72 hours, compared to control (CTRL group). (**b**) Stacked column charts show the percentage of cell cycle distribution in each phase of the cell cycle. The data shown are representative of three independent experiments.

The highest dose (100 μg/ml) of sub-5 SIO-Fl nanoparticles was selected to evaluate their effect on CaCo-2 cell cycle distribution and progression. The data showed that the cell cycle distribution was not affected upon exposure for 24 and 48 h and we did not observe any cell cycle arrest in CaCo-2 cells following exposure to the highest dose of nanoparticles even for a long period of time (72 h) (Fig. 6).

### mRNA expressions of Villin and Alkaline Phosphatase differentiation markers

**Figure 7:**
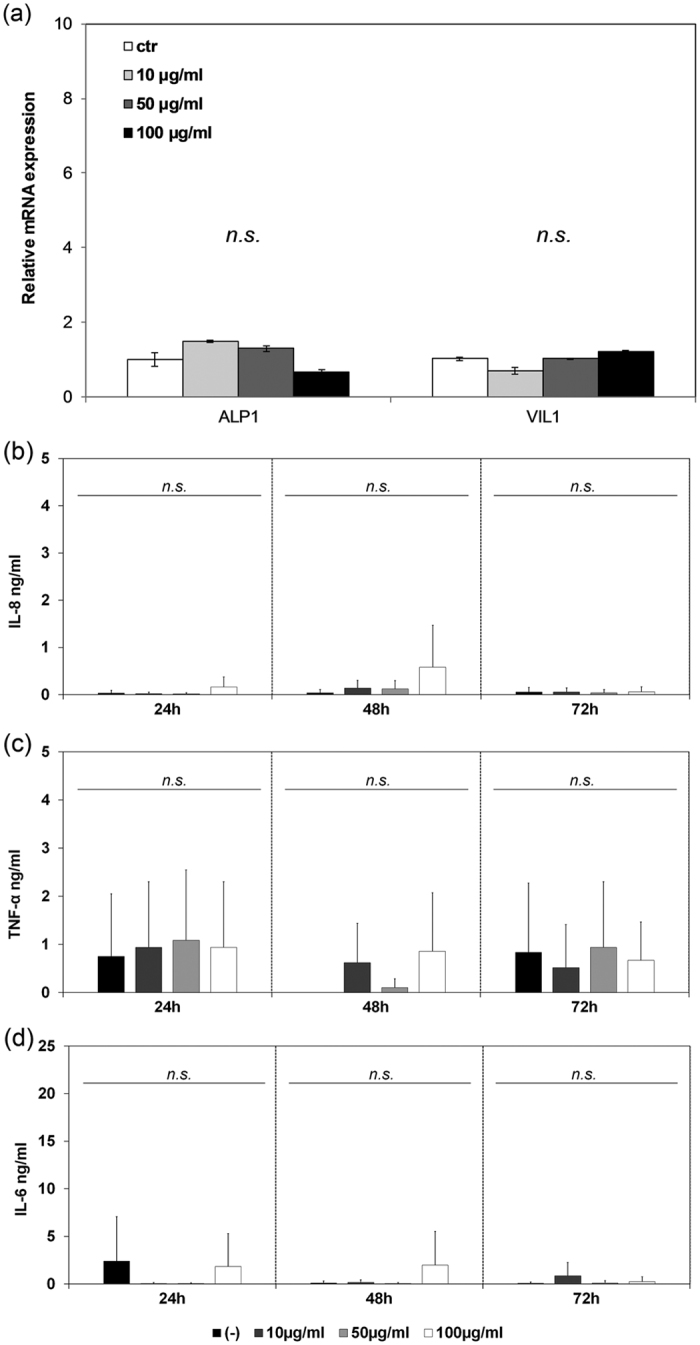
Effects of sub-5 SIO-Fl nanoparticles on expression of key differentiation markers and pro-inflammatory cytokines secretion. (**a**) qPCR analysis of key differentiation markers in CaCo-2 cells treated with 10 μg/ml, 50 μg/ml, and 100 μg/ml sub-5 SIO-Fl nanoparticles after 7 days of exposure. The expression level of VIL1 and ALP1 was analysed and resulted comparable to the control ones with no significant difference. All data were expressed as the mean ± standard deviation (SD) (n = 3) (n.s., not significant). (**b-d**) Effects of sub-5 SIO-Fl nanoparticles on the pro-inflammatory cytokines secretion in CaCo-2 cells treated with 10 μg/ml, 50 μg/ml or 100 μg/ml of nanoparticles for 24, 48 and 72 hours. Interleukin-8 (IL-8) (**b**), tumor necrosis factor α (TNF-α) (**c**) and interleukin-6 (IL-6) (**d**) levels were measured in the culture supernatant by ELISA method. All data were expressed as the mean ± standard deviation (SD) (n = 4) (*n.s*., not significant).

We examined the effect of sub-5 SIO-Fl nanoparticles on the expression of Villin (VIL1) and Alkaline Phosphatase (ALP1), both key differentiation markers of CaCo-2 cell, highly expressed in healthy growing cells. These cells undergo morphological and functional differentiation characterized by the formation of microvilli and the expression of brush border enzymes^65^, such as alkaline phosphatase, aminopeptidase N and dipeptidyl peptidase IV^66^. Alkaline phosphatase is a membrane-bound glycoprotein, which hydrolyses monophosphate esters and plays an important role in fat and the phosphate metabolism^67^,^68^,^69^. Villin, involved in the establishment of apical microvilli, is an enterocyte marker representing a morphological differentiation indicator^70^. The expression level by Real-time quantitative reverse transcriptase polymerase chain reaction analysis (qPCR) of VIL1 and ALP1 mRNAs in CaCo-2 cells treated for 7 days with three different concentrations of nanoparticles (10 μg/ml, 50 μg/ml and 100 μg/ml) resulted comparable to the control ones without significant difference (Fig. 7a). These results demonstrate that the sub-5 SIO-Fl nanoparticles do not affect the transcriptional level of VIL1 and ALP1, both involved in the differentiation process that spontaneously occurs when the CaCo-2 cells reach confluence.

### Pro-inflammatory Cytokines Secretion

In order to investigate more deeply inside the cytotoxicity effect of sub-5 SIO-Fl nanoparticles on pro-inflammatory response, CaCo-2 cells were treated with 10, 50 and 100 μg/ml nanoparticles for 24, 48 or 72 h and interleukin 8 (IL-8), tumour necrosis factor α (TNF-α) and interleukin 6 (IL-6) release was quantified in the culture supernatants by ELISA method (Fig. 7b–d). Each dose of the nanoparticles used did not affect the secretion of IL-8 by these cells at any time point (Fig. 7b). The unchanged IL-8 release after 24, 48 and 72 h of exposure suggests that there is any cellular impairment. Each dose of nanoparticles seems to induce a progressive release of TNF-α after 24 h, corresponding to about 1000 pg/ml for each concentration, followed by an overall decrease in the next 48 h of 45%, 14% and 29% respectively (72 h). Similar changes, as well, occurred in the untreated sample but, again, no significant differences are detected (Fig. 7c). TNF-α can induce CaCo-2 cells to secrete the pro-inflammatory cytokine IL-6 under appropriate condition of cellular stress. In our human cellular model, no significant differences were observed in the IL-6 release in any treated group (Fig. 7d) confirming non-activation of the oxidative stress pathway. These results lead to the conclusion that sub-5 SIO-Fl nanoparticles do not affect pro-inflammatory cytokines secretion and then do not have toxic effects in all three tested concentrations re-confirming *in vitro* biocompatibility.

## Conclusions

Recently, there has been given much emphasis on the development of hydrophobic, non-cationic cellular membrane-permeable nanoparticles and ligands. The scientific rationale for the present investigation is suggested by the need to design innovative and safe strategies to deal with human diseases. To achieve these goals, close multidisciplinary and interdisciplinary collaboration among chemists, physicists, biochemists, cell and molecular biologists was carried out.

In this work, mild condition, inexpensive and facile method, was used to synthesize ultrafine 3 nm superparamagnetic water-dispersible nanoparticles. They were obtained at room temperature and dispersed in alkaline aqueous solution using environmentally friendly and biocompatible reagents.

The nude ultrafine 3 nm superparamagnetic nanoparticles were covered with silica and labelled with fluorescein through a new one pot synthetic strategy modifying procedures already reported^48^,^49^,^50^.

We demonstrate that sub-5 nm silica-coated magnetic iron oxide fluorescent nanoparticles are internalized and, even at the highest concentration used, are biocompatible, non-toxic, do not interfere with the cell cycle distribution nor with the expression of key differentiation markers and do not affect pro-inflammatory cytokines response in CaCo-2 cells.

Since their magnetic nature (average core size 3 nm), our nanoparticles could be easily *in vivo* directed toward the desired tissues/organs to shuttle drugs upon the application of an external static magnetic field. They could be used as efficient vehicles for drug/gene delivery for antiblastic therapies, enhancing the efficacy of treatments with reduced systemic toxicity. Moreover, these nanoparticles can maintain the ability to act as antennae in an external alternating magnetic field to convert electromagnetic energy into heat, in order to synergize the action of the shuttled drugs with hyperthermia.

For their tiny dimension and magnetic properties these nanoparticles could have advantages in diagnostic applications over larger nanoparticles and promise their safe employ for innovative nanomedicine applications through improvement of cell delivery with the aim to potentiate the regeneration of damaged tissues.

## Methods

### Chemicals

Iron(III) nitrate nonahydrate (Fe(NO_3_)_3_ 9H_2_O, ferrous chloride (FeCl_2_·4H_2_O), sodium nitrate (NaNO_3_), ammonium nitrate (NH_4_NO_3_), tetramethylammonium nitrate ((CH_3_)_4_N(NO_3_)), sodium hydroxide (NaOH), ammonia solution (NH_4_ OH, 30%), tetramethylammonium hydroxide (N (CH_3_)_4_OH), aminopropyltriethoxysilane (H_2_N(CH_2_)_3_Si(OC_2_H_5_)_3_, APS), tetraethoxysilane (CH_3_CH_2_OSi (OCH_2_CH_3_), TEOS) and fluorescein isothiocyanate (FICT) were purchased from Sigma-Aldrich and used without further purification.

### One-pot synthetic procedure

The sub-5 nm silica-coated magnetic iron oxide fluorescent nanoparticles were obtained modifying reported procedures^48^,^49^,^50^ by covalently attaching the FICT dye to the coupling agent APS, due to the addition reaction of the amine with the thioisocyanate group.

Typically, an ethanol solution (5 ml) of FICT (11 mg) and APS (11 mg) was prepared and slowly added to ethanol suspension of magnetite nanoparticles (above described), obtained mixing anhydrous ethanol (80 ml) with ammonium hydroxide (30%, 8, 5 ml), TEOS (2, 8 ml) and 20 mg of magnetite nanopowder. Water was excluded to prevent hydrolysis and condensation of the silane molecules. The dye nanocomposite was collected by drying after centrifugation (8000 rpm, 5 min).

### Nanoparticles characterizations

#### TEM measurements

TEM measurements were carried out using a Philips CM120 microscope operated with an accelerating voltage of 80–100 keV. The nanoparticles were dispersed on commercially-available copper-carbon formvar TEM grids. The statistical analysis of nanoparticle sizes was performed using SPIP^TM^ software on raw image data.

#### Dynamic Light Scattering and Zeta potential measurements

Solutions were characterized with Dynamic Light Scattering with Zetasizer Nano ZS (Malvern, Herrenberg, Germany) equipped with a 633 nm He–Ne laser and operating at an angle of 173°. Solvent-resistant micro cuvettes (ZEN0040, Malvern, Herrenberg, Germany) have been used for experiments with a sample volume of 40 μl. The measurements were performed at a fixed position (4.65 mm) with an automatic attenuator and at a controlled temperature (20 °C). For each sample, five measurements were averaged, the diffusion coefficient D has been retrieved from autocorrelation functions. The equivalent Hydrodynamic Diameter (D_H_) was obtained by the Stokes-Einstein equation. Data analysis was performed by Malvern Zetasizer software^71^. The ζ-potential was calculated from the electrophoretic mobility by means of the Henry correction to Smoluchowski’s equation with Data analysis was performed by Malvern Zetasizer software. Zetasizer Nano ZS (Malvern, Herrenberg, Germany) averaging 5 measurements^72^.

### Fourier transform infrared spectroscopy study (FTIR)

FTIR measurements were performed by means of a VECTOR 22 (Bruker) FTIR interferometer equipped with a DTGS detector. The investigated samples, namely pristine fluorescein isothiocyanate (Fl-NCS) and sub-5 nm silica-coated magnetic iron oxide fluorescent (sub-5 SIO-Fl) nanoparticles, were mixed with dried KBr and pressed into pellets (diameter 1 cm, thickness 1 mm).

### Biological analyses

#### Cell culture

The human colon carcinoma CaCo-2 cell line, obtained from the American Type Culture Collection (ATCC, HTB-37 Rockvile, MD, USA) was grown in high-glucose Dulbecco’s modified Eagle’s Medium (DMEM; Euroclone) supplemented with 10% heat-inactivated foetal bovine serum (FBS, Euroclone), 2 mM L-glutamine (Sigma), 1.0 unit/ml penicillin (Sigma), and 1.0 mg/ml streptomycin (Sigma). The cells were seeded at a concentration of 2 × 10^4^ cells/cm^2^ and cultured at 37 °C in a humidified incubator containing 5% CO_2_. Whenever CaCo-2 cells were treated with nanoparticles, the sub-5 SIO-Fl nanoparticles were subjected to ultrasonication for 5 min to break up aggregation, mixed into cell culture medium at the indicated concentrations and added to the cells 24 h after seeding.

#### Uptake studies

At 1, 2, 4, 6, 10, 24 and 48 h after exposure with 10 μg/ml, 50 μg/ml and 100 μg/ml of nanoparticles, the cell medium was discarded to remove the sub-5 SIO-Fl nanoparticles not up taken. The cells were then repeatedly washed with PBS and analysed with an ELISA reader (VICTOR^3^V, PerkinElmer).

The time-dependent uptake of sub-5 SIO-Fl nanoparticles by CaCo-2 cell has been detected measuring the relative fluorescence emitted at 485/535 nm from the sub-5 SIO-Fl nanoparticles up taken by CaCo-2 cells at the indicated time points.

#### Confocal Laser Scanning Microscopy

48 h after exposure with 50 μg/ml of nanoparticles, cells cultured on 0.01% poly-lysine-treated glass cover slips were repeatedly washed with PBS and fixed in 4% paraformaldehyde for 10 min, rinsed twice with PBS, permeabilized with PBS containing 1% bovine serum albumin (PBS/BSA) and 0.2% triton X-100 for 5 min, and rinsed again in PBS. The cells were also incubated with phalloidin tetramethylrhodamine isothiocyanate conjugated (1:100), an anti-actin toxin (Sigma) in a blocking buffer for 1 h^73^, rinsed three times in PBS/BSA, and finally stained for nuclei localization with Hoechst 33342. Cover slips were assembled, cell-side down, on a microscope slide with 0.625% N-propyl gallate in PBS glycerol 1:1. The cover slip “sandwich” was sealed to prevent exposure to air and to exclude and prevent the crystal formation of H_2_O. The fluorescence analyses were performed by using LEICA TCS 4D Confocal Microscope supplemented with an Argon Krypton laser and equipped with 40 × 1.00–0.5 and 100 × 1.3–0.6 oil immersion lenses. Confocal optical Z sections were acquired at 2-μm intervals for each field considered and a middle confocal Z section is shown.

#### Cell metabolic activity assay (WST-1)

Cell viability of CaCo-2 cells, treated at three nanoparticle concentrations (10 μg/ml, 50 μg/ml and 100 μg/ml), was studied by quantification of cell metabolic activity using the Water Soluble Tetrazolium Salt (WST-1) test, a colorimetric assay based on oxidation of tetrazolium salts (Roche Diagnostics, Basel, Switzerland). WST-1 reagent, diluted 1:10, was added to the medium at day 1, 2, 3, 4 and 7 of cell culture and after 2 h of incubation in a humid atmosphere, the cells were analysed by formazan salt formation. The formazan quantification was performed by measuring the absorbance at 450 nm with an ELISA reader (Biotrack II; Amersham Biosciences, Little Chalfont, UK). Cell viability was reported as the percentage of the absorbance of sub-5 SIO-Fl nanoparticles treated cells in relation to the absorbance of untreated cells.

#### Cell proliferation (BrdU) analysis

Cell proliferation of CaCo-2 cells, treated at three nanoparticle concentrations (10 μg/ml, 50 μg/ml and 100 μg/ml), was evaluated by Bromodeoxyuridine (BrdU) incorporation assays. 10 mM Bromodeoxyuridine was added to the cell medium at day 1, 2, 3, 4 and 7 and maintained for 18 hours. Cells were then fixed and incubated for 30 min at room temperature with anti-BrdU antibody (Cell Proliferation Kit; Roche Diagnostics). After incubation with 2,20-Azino-bis (3-ethylbenzothiazoline-6-sulfonic acid) for 30 min the absorbance of cell supernatant was measured with an ELISA reader at 450 nm.

#### Cell cycle analysis by flow cytometry

Cell cycle was evaluated after 24, 48 and 72 h of exposure with 100 μg/ml of nanoparticles by flow cytometry analysis. To obtain a cell suspension without clumps, cells were detached with trypsin, washed ones in cold FACS buffer (2 mM EDTA, 0.5% FBS in PBS 1x), and twice in PBS, suspended in 1 ml PBS and fixed in 10 ml 70% cold ethanol at 4 °C. Fixed cells were washed in PBS, then stained with propidium iodide (20 ug/mL; Sigma) and RNase A (250 ug/mL; Sigma) solution for 30 min at room temperature in the dark. About 1 × 10^6^ cells were acquired using a FACSCalibur (Becton Dickinson) cytometer and the cell cycle analysis was performed by ModFIT LT 2.0 software.

#### Real-time quantitative reverse transcriptase polymerase chain reaction analysis (qPCR)

Total RNA was extracted from untreated and sub-5 SIO-Fl nanoparticles treated CaCo-2 cells using TRIzol Reagent (Invitrogen) after 7 days of cell culture. One microgram of total RNA was used to synthesize first strand cDNA with random primers, using 100 U of ImProm-II TM RT–PCR kit (Promega, Madison, WI, USA) according to the manufacturer. Quantification of all gene transcripts was carried out by real-time quantitative reverse transcriptase polymerase chain reaction (qPCR). It was also carried out in the absence of reverse transcriptase to check for genomic DNA amplification. Experiments were carried out to contrast relative levels of each transcript and endogenous control GAPDH in every sample. The data were analysed using the equation described by Livak^74^ (details are provided in the Supplementary Methods).

#### ELISA assay

Supernatants of CaCo-2 cells, treated with 10 μg/ml, 50 μg/ml and 100 μg/ml of sub-5 SIO-Fl nanoparticles, were collected at 24, 48 and 72 h, centrifuged at 1200 rpm for 5 min and stored at −80 °C until use. Interleukin-6 (IL-6), tumour necrosis factor-α (TNF-α) and interleukin-8 (IL-8) cytokines release was measured in the culture medium using ELISA kits (PeproTech^®^ EC Ltd., UK) according to the manufacturer’s instructions. Cell supernatants and human recombinant standards were serially diluted in 1 × PBS/0.05% Tween-20/0.1% BSA, and added to the microplates. Interleukins binding were detected by biotin-avidin detection step, followed by chromogen 2,2′-azino-bis(3-ethylbenzothiazoline-6-sulphonic acid (ABTS) (BioVision, Inc., Milpitas, CA, USA) or 3,3′,5,5′-Tetramethylbenzidine (TMB) (Enzo Life Sciences, Inc., Farmingdale, NY, USA) incubation. Colour development was monitored at 405 or 450 nm. The concentration of cytokines in the samples was determined from the standard curve.

#### Statistical analysis

MedCalc software was used for statistical analysis, and the significance level adopted for all analyses was P < 0.05.

For the results of cellular viability, cellular proliferation and cellular uptake data were analysed by repeated measures analysis of variances ANOVA-test (Time × Treatment with time as a repeating variable), followed by one-way ANOVA test at each day point to verify the statistical significance among different groups. For RT-qPCR analysis and Cytokines Secretion study, data were analysed by one-way ANOVA test.

## Additional Information

**How to cite this article:** Foglia, S. *et al. In vitro* biocompatibility study of sub-5 nm silica-coated magnetic iron oxide fluorescent nanoparticles for potential biomedical application. *Sci. Rep.* **7**, 46513; doi: 10.1038/srep46513 (2017).

**Publisher's note:** Springer Nature remains neutral with regard to jurisdictional claims in published maps and institutional affiliations.

## Supplementary Material

Supplementary Information

## Figures and Tables

**Figure 1:**
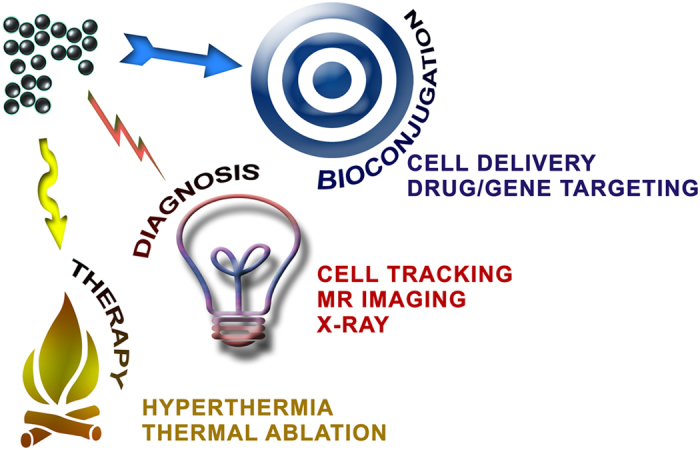
A schematic representation of some of the unique and advantageous applications of superparamagnetic iron oxide nanoparticles (IONPs).

**Table 1:** FTIR peak position (cm^−1^) and assignment for sub-5 SIO-Fl and pristine Fl-NCS.

Experimental results	Fl-NCS	Assignment
sub-5 SIO-Fl		
565		Fe-O stretching
1035		Si-OH, Si-O-C stretching
1110		asymmetric Si-O-Si stretching
	1113	Aromatic C-H bending
1195	1173	CCH bending + phenolic C-OH
1200	1207	XR C-O-C stretching
1266	1264	Carboxyl C-O stretching
1302	1308	Phenoxide stretching conjugated with XR stretching
1330		XR C-C stretching
1384	1386	Symmetric COO^−^ stretching
1466 sh	1458	XR C-C stretching conjugated with COO^−^ stretching
1499	1490 sh	central ring breathing C-C stretching
1557	1539	XR C-C stretching
	1590	Asymmetric COO^−^ stretching
1635 sh		O-H and/or N-H bending
1720	1740	Carboxyl C = O stretching
	2020	NCS stretching

Sh refer to shoulder; XR is abbreviation of xanthene ring.
