# Supplementary material for: In vitro biocompatibility study of sub-5 nm silica-coated magnetic iron oxide fluorescent nanoparticles for potential biomedical application
Source: Sci Rep. 2017 Apr 19;7:46513. doi: 10.1038/srep46513 (PMC5395943; doi:10.1038/srep46513)
Supplement: Supplementary Information [file srep46513-s1.pdf]

In vitro biocompatibility study of sub-5nm silica-coated magnetic iron oxide fluorescent nanoparticles for potential biomedical application

Sabrina Foglia,[1a] Mario Ledda,[2a] Daniela Fioretti,[2] Giovanna Iucci,[3] Massimiliano Papi,[4] Giovanni Capellini,[3] Maria Grazia Lolli,[2] Settimio Grimaldi,[2] Monica Rinaldi,[2b] and Antonella Lisi[2b*]

[1]Institute of Materials for Electronics and Magnetism (IMEM), Department of Engineering, ICT and technologies for energy and transportation, National Research Council (CNR), Parma, Italy
[2]Institute of Translational Pharmacology (IFT), Department of Biomedical Sciences, National Research Council (CNR), Rome, Italy
[3]Department of Science, University Roma Tre, Rome, Italy
[4]*Institute of Physics*, Catholic University of the Sacred Heart, Rome, Italy
[a]These authors shared first authorship
[b]These authors shared senior authorship
Correspondence and requests for materials should be addressed to A.L. (email: antonella.lisi@ift.cnr.it)

Supplementary Methods

Nanoparticles synthesis

Magnetite nanoparticles were synthesized by procedure reported in Vayssières *et al.*, [1] using simpler co-precipitation of ions Fe^{2+} and Fe^{3+} in a solution containing a mixture of $Fe(NO)_3$ and $FeCl_2$ and $NaNO_3$, in a 3M $NaNO_3$ solution. The pH of the medium was kept constant in a range from 10 to 12, by addition of the corresponding base (NaOH). The reactions were performed at room temperature, controlled atmosphere, with continuous bubbling of nitrogen. Fe_3O_4 nanopowder was collected after centrifugation (11000 rpm, 10 min) and dried under dynamic vacuum.

1. Vayssieres, L., Chaneac, C., Tronc, E. & Jolivet, J. P. Size Tailoring of Magnetite Particles Formed by Aqueous Precipitation: An Example of Thermodynamic Stability of Nanometric Oxide Particles. *J. Colloid Interface Sci.* **205**, 205-212 (1998).

X-ray diffraction analysis (XRD)

XRD measurements were performed using a powder diffractometer (Philips X'Pert Pro) operating in the reflection mode with CuKa radiation and equipped with a graphite back monochromator.

Magnetization measurements

The magnetic property of the nanoparticles was analyzed by vibrating sample magnetometry (VSM) and superconducting quantum interference device (SQUID) at a magnetic field of 100 Oe for both field-cooling (FC) and zero-field-cooling (ZFC).

Phase contrast and Fluorescence Microscopy

The CaCo-2 cells ($2x10^4$ cells/cm^2) were seeded on cover glasses in a 24-well plate and allowed to adhere for 24h. Then sub-5 SIO-Fl nanoparticles, treated ultrasonically for 5 min to break up aggregation, were dispersed in cell culture medium and added to the CaCo-2 cells at a concentration of 10 µg/ml, 50µg/ml and 100 µg/ml. 48h after exposure the cells were repeatedly washed with PBS and fixed in paraformaldehyde 4% at 4°C for 10 min, then washed twice in Ca^{2+}/Mg^{2+}-free PBS and stained for nuclei localization with Hoechst 33342 (trihydrochloride trihydrate). Phase contrast and fluorescence measurements were performed by using an inverted microscope (Olympus IX51, RT Slider SPOT - Diagnostic instruments, Sterling Heights, MI, USA) equipped

with a 20X, 40X and 60X objective and with a cooled CCD camera (Spot RT Slider, Diagnostic Instruments).

Fluorescence microscopy analysis of Rhodamine phalloidin-labelled F-actin

The CaCo-2 cells ($2x10^4$ cells/cm^2) were seeded on cover glasses in a 24-well plate and after 24 h of culture incubated with sub-5 SIO-Fl nanoparticles at three different concentrations (10µg/ml, 50µg/ml, and 100 µg/ml). 7 days after exposure the cells were repeatedly washed with PBS and fixed in paraformaldehyde 4% at 4°C for 10 min, washed twice in Ca^{2+}/Mg^{2+}-free PBS and permeabilized at room temperature for 15 min (0.1% Triton X-100, 1% BSA; Sigma-Aldrich). The cells were also incubated with phalloidin tetramethylrhodamine isothiocyanate conjugated (1:100), an anti-actin toxin (Sigma), in a blocking buffer for 1 h. Cells were washed three times with PBS, counterstained for nuclei localization with Hoechst 33342 (trihydrochloride–trihydrate), and examined. Fluorescence measurements were using an inverted microscope (Olympus IX51, RT Slider SPOT - Diagnostic instruments, Sterling Heights, MI, USA) equipped with a 20X, 40X and 60X objective and with a cooled CCD camera (Spot RT Slider, Diagnostic Instruments).

Real-time quantitative reverse transcriptase polymerase chain reaction analysis (qPCR)

Amount of target was calculated using the $2^{-\Delta\Delta Ct}$ equation.

$^{\Delta}Ct$ = (average target Ct – average GAPDH Ct)

$^{\Delta\Delta}Ct$ = (average $^{\Delta}Ct$ treated sample – average $^{\Delta}Ct$ untreated sample)

Before using $^{\Delta\Delta}Ct$ method for quantification, we performed a validation experiment to demonstrate that efficiency of target genes and reference GAPDH were equal. Real-time PCR was performed with Sybr Green I Mastermix (Applied Biosystems) using an ABI PRISMTM 7000 Sequence Detection System. Each reaction was run in triplicate and contained 0,5 µl of cDNA template along with 250 nM primers in a final reaction volume of 25 µl. The genes investigated were ALP1 and VIL1. The specific primers used are the following:

ALP1: 5’-ccaggacatcgccactcag-3’; 5’-tcagtgcggttccacacata-3’

VIL1: 5’-gctatctatggtgtgggaag-3’; 5'-cctgtagtctcttggtgttg-3’

GAPDH: 5’-catcatctctgccccctct-3’; 5’-caaagttgtcatggatgacct-3’

Cycling parameters were 50°C for 2 min., then 95°C for 10 min. in order to activate DNA polymerase, then 40 cycles of 95°C for 15 seconds and finally 60°C for 1 min. The Melting curves were performed using Dissociation Curves software (Applied Biosystems) to ensure that only a single product was amplified. As negative controls, we used tubes where RNA or reverse transcriptase was omitted during the RT reaction.

TEM analysis

The SIO nanoparticles, treated ultrasonically for 5 min to break up aggregation, were dispersed in cell culture medium and added to the CaCo-2 cells at a concentration of 50µg/ml. After 1h exposure the cells were repeatedly washed with PBS and fixed using glutaraldehyde (2.5%) for 1 h. The cells were washed several times with 0.15M cacodylate buffer and post-fixed with 1.5% osmium tetroxide for 1 h. After further washing with cacodylate buffer, the samples were dehydrated through an ethanol gradient from 70% to 100%, embedded and cut into ultrathin sections and examined as previously described.

Supplementary Figures

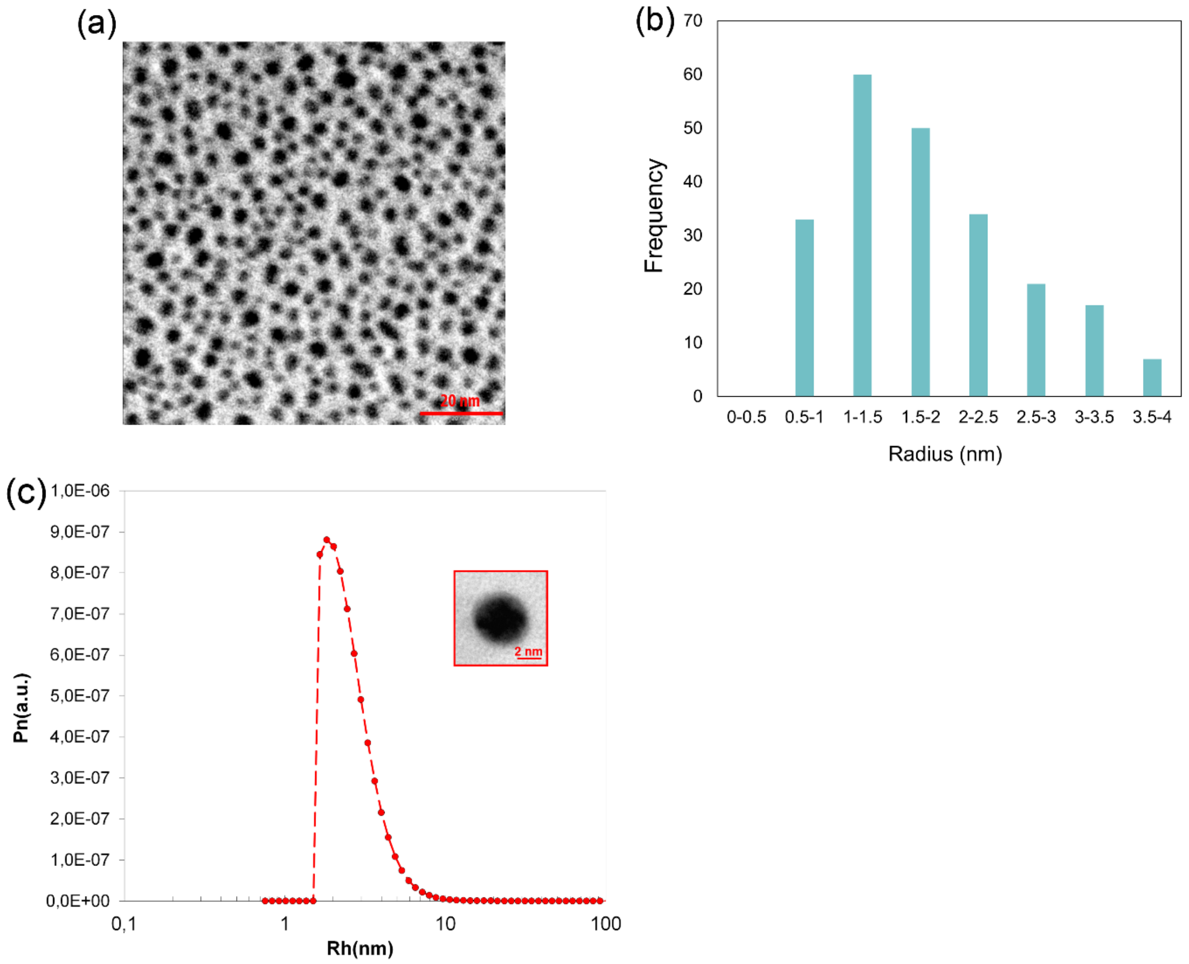

Supplementary Figure S1. Characterization of 3 nm ultrafine superparamagnetic nanoparticles. Representative TEM image of spherical 3 nm ultrafine superparamagnetic nanoparticles (a). 3 nm ultrafine nanoparticles size distribution obtained from the TEM images (sample size=220 nanoparticles) (b) and from DLS (c). A representative high-resolution image of a single 3 nm ultrafine nanoparticles is reported in the (c) inset.

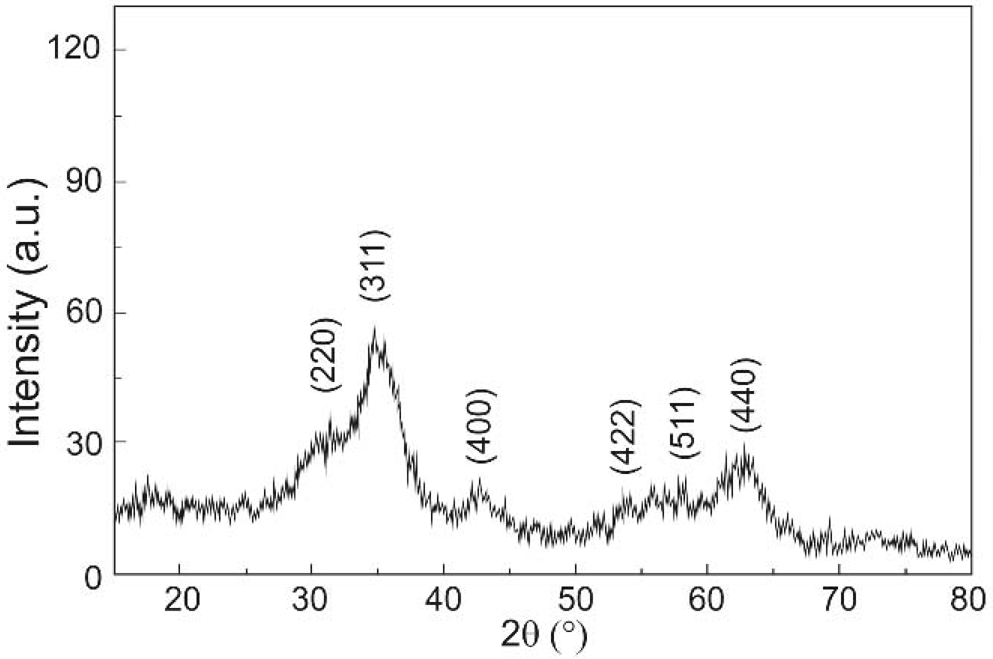

Supplementary Figure S2. X-ray diffraction pattern of 3 nm ultrafine nanoparticles.

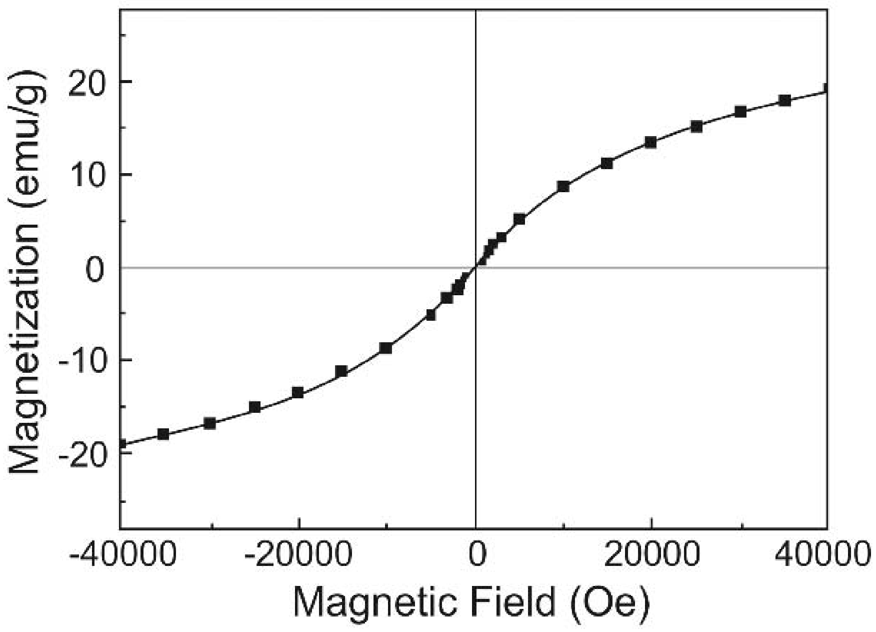

Supplementary Figure S3. Magnetization curve of 3 nm ultrafine nanoparticles obtained by VSM at room temperature.

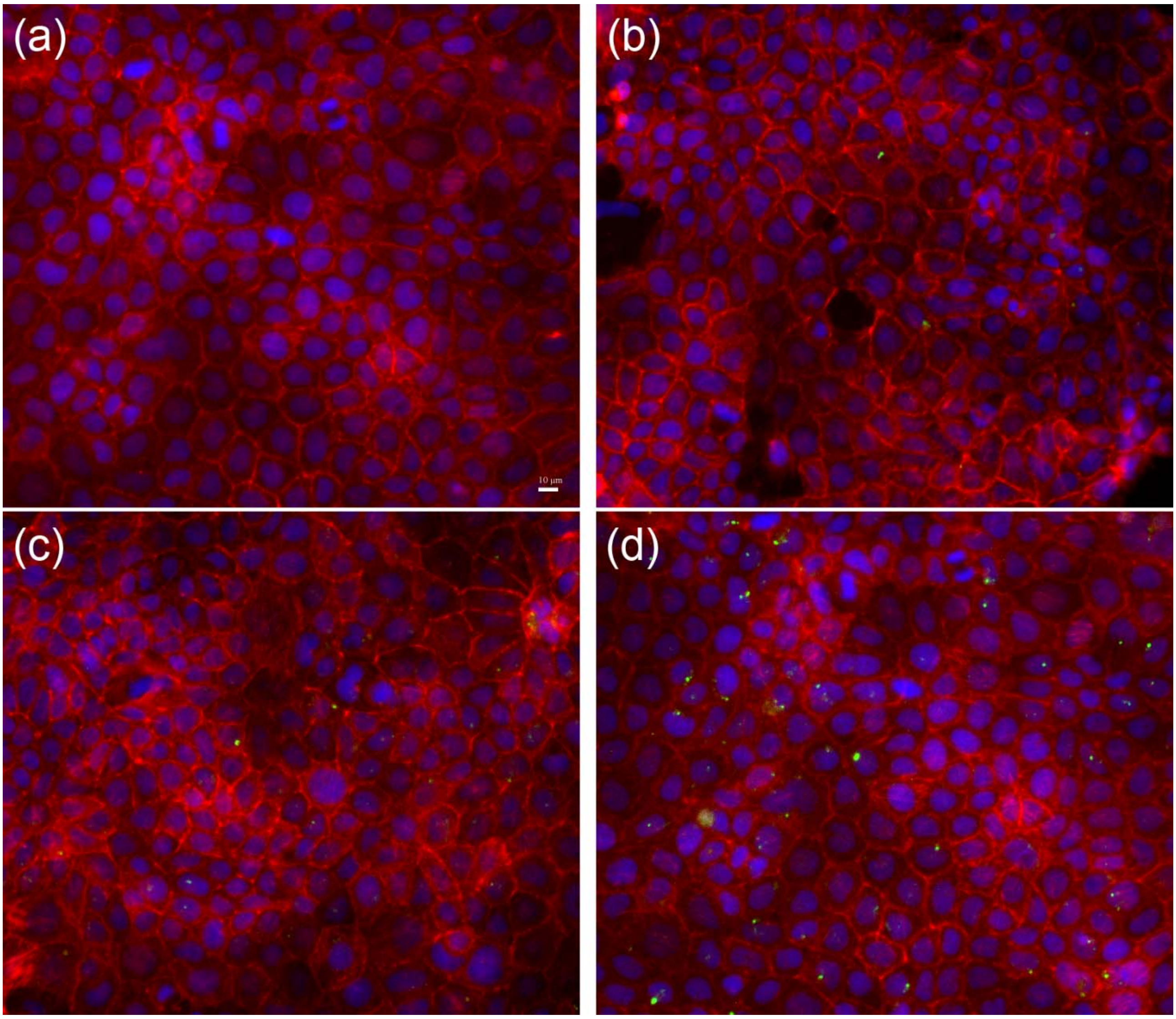

Supplementary Figure S4. Fluorescence microscopy analysis of rhodamine phalloidin-labelled F-actin (red) of CaCo-2 cells. Untreated cells (a) and cells treated with 10µg/ml (b), 50 µg/ml (c) and 100 µg/ml (d) of sub-5nm silica-coated magnetic iron oxide fluorescent nanoparticles (green) after 7 days of exposure. The sub-5 SIO-Fl nanoparticles inside the cells are shown and do not interfere with the cytoskeletal organization. Nuclei are counterstained with Hoechst (blue). Photographs were taken at a magnification of 20X.

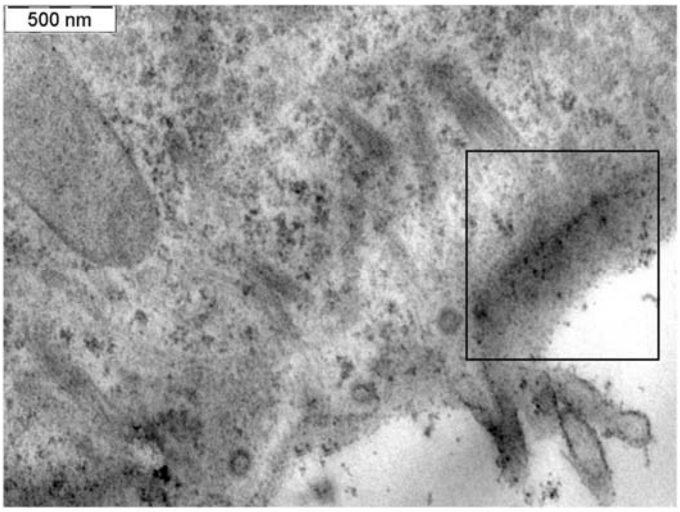

Supplementary Figure S5. TEM image of Caco-2 cells after 1h of exposure with SIO nanoparticles (50µg/ml). The box highlights the iron oxide nanoparticles within Caco-2 cells.

Supplementary Table

Time (h)	1	12	24	36	48	60
D_H (nm)	8.5±2.2	9.4±2.8	9.5±2.8	9.3±2.7	9.5±3.1	9.4±2.9
ζ (mV)	-15.5±3.4	-9.2±3.1	-9.2±2.8	-9.1±3.0	-9.1±3.1	-9.2±3.4

Supplementary Table S1. Dynamic Light Scattering and Zeta potential measurements of SIO nanoparticles after exposure to complete cell culture medium.
